# Assessing the impact of an environmentally friendly approach on irreversible dental hydrocolloid performance

**DOI:** 10.1038/s41598-024-83035-w

**Published:** 2024-12-16

**Authors:** Leonie Beuter, Christoph Bourauel, Lamia Singer

**Affiliations:** 1 Oral Technology, Dental School, Medical Faculty, University Hospital Bonn, Bonn, Germany; 2 Department of Orthodontics, Dental School, Medical Faculty, University Hospital Bonn, Bonn, Germany

**Keywords:** Irreversible hydrocolloid gels, Green synthesis, Antimicrobial activity, Nanotechnology, *Syzygium aromaticum*, *Zingiber officinale*, Microbiology, Plant sciences, Medical research

## Abstract

Background: Impression materials can harbour microorganisms from saliva and blood, posing cross-contamination risks. However, post-setting disinfection might compromise the dimensional accuracy and mechanical properties of alginates. Hence, it was the aim of this research to assess the detail reproduction, tear strength, elastic recovery, and surface quality of the gypsum model of newly developed dental alginates with inherent antimicrobial properties. Methods: Three dental alginate groups with antimicrobial alterations were formulated. One group replaced water with 0.2% chlorhexidine solution (CHX group), while the other two utilized water-based extracts of *Syzygium aromaticum* (*SA*, clove) or *Zingiber officinale* (*ZO*, ginger) to reduce silver nitrate, resulting in two silver nanoparticles (AgNPs)/extract (clove or ginger) mixture solutions. These mixture solutions were employed for the preparation of dental alginate yielding the *SA* + AgNP and *ZO* + AgNPs groups. All modified groups were compared to an unmodified control group that used water for mixing. Elastic recovery, detail reproduction, and tear strength were assessed following the ISO 21563:2021 standard. The surface roughness of plaster models was analysed using the optical profilometer. Elastic recovery was assessed by applying and then releasing load on alginate specimens to measure their ability to recover from deformation. Detail reproduction was evaluated by observing the reproducibility of a 50 μm line in a metallic mold using a light microscope, while tear strength was determined by stretching the specimens until failure at a constant speed of 500 mm/min. Results: All tested groups exhibited elastic recovery values meeting ISO standards for hydrocolloid impression materials. Regarding detail reproduction, both the control and modified alginates successfully reproduced the 50-µm line without interruption in all specimens. Tear strength values for all tested groups remained within the acceptable documented ranges, surpassing the minimum requirement of 0.38 N/mm as mandated by ISO 21563:2021. The *ZO* + AgNPs (0.94 ± 0.17 N/mm) demonstrated significantly higher tear strength values and surface roughness values compared to the other tested groups. Conclusions: Chlorhexidine, *Syzygium aromaticum*, and *Zingiber officinale* green-synthesized silver nanoparticles are promising, cost-effective alternatives for disinfecting alginate impressions without compromising performance. Green nanoparticle synthesis is a safe, efficient, and non-toxic method, potentially synergizing metal ions with plant extract.

## Introduction

Alginate impression material holds significant importance in dentistry as a widely used and cost-effective material for creating dental impressions. It is the standard choice among dentists due to its ease of use, ability to capture fine details, and patient comfort during the impression-making process. Alginate is a polysaccharide derived from the cell walls of brown algae belonging to the Phaeophyceae family^1^. When water is added to the alginate powder, it undergoes a transition into a gel, which solidifies into an irreversible elastic material^2,3^. The powder contains fillers (mostly silica), soluble sodium alginate, calcium sulfate as a reactor, fluorides to accelerate the setting process, trisodium phosphate as a retarder, and colour and flavour additives^4^.

The reaction of sodium alginate with calcium sulfate leads to the gelation of alginate. The reaction results in the formation of calcium alginate gel, along with the production of sodium sulfate^5^. Initially, the retarder trisodium phosphate reacts with calcium sulfate, forming calcium phosphate. This reaction delays the setting reaction, and the consumption of trisodium phosphate can trigger the setting process^3^. Dentists commonly use alginate impressions for fabricating study models, orthodontic appliances, and provisional restorations^2^. However, alginate has limitations, including its dimensional instability over time and susceptibility to deformation if not handled and stored properly^6^. Despite these drawbacks, its convenience, affordability, and adequate accuracy for many dental applications maintain its widespread use in dental practices^6^.

Disinfection of alginate impressions in dentistry is crucial to prevent cross-contamination and ensure patient safety^7^. Developing effective methods for disinfection without compromising the integrity and dimensional stability of the alginate material remains an ongoing challenge in dental practice^8^. Alginate’s hydrophilic nature poses challenges during disinfection. Furthermore, alginate’s porosity allows microorganisms to penetrate and remain trapped within its structure, making complete disinfection difficult to achieve. Post setting disinfectants might not effectively reach all areas, especially deep undercuts, potentially leaving pathogens behind^9^.

Incorporating disinfectants like silver nanoparticles into alginate material presents a promising approach for enhancing intrinsic disinfection in dental impressions^10,11^. Silver nanoparticles possess potent antimicrobial properties, effectively targeting and eliminating various microorganisms. When integrated into alginate, these nanoparticles can potentially penetrate the material’s porous structure, reaching deeper areas that conventional disinfectants may struggle to access^10,11^.

In the traditional synthesis of silver nanoparticles, the bottom-up method and the top-down method are two possible approaches. In the bottom-up approach, nanoparticles are built from atoms and molecules, increasing the size to the size of a nanoparticle. In contrast, the top-down approach involves techniques to break down a bulk material into smaller fragments, decreasing the size to achieve nanoparticles^12^. Common manufacturing methods have been predominantly chemical or physical, which, however, are considered environmentally unfriendly because of their high-energy requirements, and potential formation of toxic by-products. Another drawback is their high production costs^13^.

Due to these limitations, green synthesis of nanoparticles using medicinal plants has emerged as an eco-friendly and sustainable method to produce silver nanoparticles (AgNPs). Plant extracts contain various phytochemicals acting as reducing and capping agents, facilitating the reduction of silver ions to nanoparticles^14^. These extracts not only serve as reducing agents but also contribute additional antimicrobial compounds, augmenting the overall antimicrobial potency of the synthesised nanoparticles.

This approach harnesses the bioactive potential of plants while promoting a greener and cost-effective means to create antimicrobial agents with broad applications in healthcare and numerous other fields^15,16^. Silver nanoparticles generated by using plant extracts are less toxic compared to those synthesised through chemical methods. A synergistic effect between silver nanoparticles and phytomolecules present in the plant extracts results in a greater therapeutic effectiveness^17^.

The exploration of generating silver nanoparticles using a green synthesis method employing plant extracts from *Syzygium aromaticum* (*SA*, clove) and *Zingiber officinale* (*ZO*, ginger) initiated the objective of this investigation. In our earlier research, we tested these two modifications of alginate for their antimicrobial effectiveness, comparing them to negative (unmodified) and chlorhexidine-positive controls^18^. The results showed spherical nanoparticles, with the ginger extract producing clusters ranging from 400 nm to 1 μm, while the clove extract resulted in smaller nanoparticles (50–100 nm) with increased aggregation. Energy Dispersive X-ray Spectroscopy (EDX) confirmed the presence of silver nanoparticles and clusters, as well as potassium, sodium, sulfur, magnesium, phosphorus, oxygen, and chlorine. The modifications involving CHX, *ZO* + AgNPs, and *SA* + AgNPs exhibited significantly enhanced antimicrobial activity against various strains, such as *Candida albicans* (*C. albicans*), *Streptococcus mutans* (*S. mutans*), methicillin-resistant Staphylococcus aureus (MRSA), and methicillin-sensitive Staphylococcus aureus (MSSA), when compared to the control group. This improvement was attributed to the presence of several identified organic compounds, including terpenes, phenolic acids, and flavones, detected through chemical analysis in both extracts. Thus, these compounds not only acted as reducing and stabilising agents but also demonstrated antimicrobial properties during the green synthesis of AgNPs. Furthermore, the dimensional accuracy of both AgNPs modified groups was similar to that of the unmodified negative control. However, the CHX group showed significantly altered dimensional changes in comparison to the negative control group^18^.

Expanding upon our established findings and recognising the synergistic antimicrobial effects between metal ions and phytotherapeutic agents found in *Syzygium aromaticum* and *Zingiber officinale* extracts, this study aimed to further evaluate our modifications. Specifically, we assessed in the current study the elastic recovery, detailed reproduction, tear strength, and surface roughness of gypsum models. The objective was to determine whether the modifications negatively affected the properties of the alginate. The null hypothesis for this study is that the incorporation of antimicrobial agents, specifically chlorhexidine or silver nanoparticles derived from *Syzygium aromaticum* (clove) and *Zingiber officinale* (ginger), will not cause any significant differences in the detail reproduction, tear strength, elastic recovery, or surface quality of dental alginates when compared to the unmodified control group that uses water for mixing.

## Materials and methods

Comprehensive list of all materials utilised in the study, along with their respective purposes and brand names are listed in Table 1.

**Table 1:** Materials used in the Experiments.

Material	Brand	Use
Dental Alginate	Blueprint Xcreme^®^ (Dentsply DeTrey GmbH, Kostanz, Germany)	Dust-free, normal-setting dental alginate is used as the base material, subsequently modified with antimicrobial agents.
Chlorhexidine digluconate solution	Sigma-Aldrich, St. Louis, MO, USA 18472-51-0	Mixed with distilled water to create a solution for alginate modification as an antimicrobial agent.
Silver nitrate powder	Sigma-Aldrich, St. Louis, MO, USA 209139-25G	≥ 99% purity; mixed with distilled water for alginate modification to synthesise silver nanoparticles.
Clove buds (*Syzygium aromaticum*)	Commercially available	Used for the preparation of aqueous plant extracts for the synthesis of silver nanoparticles.
Ginger roots (*Zingiber officinale*)	Commercially available	Utilised for the preparation of aqueous plant extracts used in the synthesis of silver nanoparticles.
Gypsum powder	BonStone^®^ Super, Wiegelmann Dental GmbH, Bonn, Germany)	Employed in creating gypsum test models.

### Preparation of modification solutions and experimental groups

The modification solutions were prepared sequentially. Initially, a 0.2% chlorhexidine solution was created by mixing 1 g of chlorhexidine digluconate with 500 ml of distilled water to modify the alginate. Subsequently, to carry out the green synthesis of silver nanoparticles, a 0.2% silver nitrate solution was formed by dissolving 1 g of silver nitrate powder in 500 ml of distilled water. This resulting solution was then combined with aqueous extracts obtained from cloves (*Syzygium aromaticum*) or ginger roots (*Zingiber officinale*) at a given ratio.

To obtain the aqueous extract of *Syzygium aromaticum*, clove buds were dried, pulverised, and soaked in a solution of 80% distilled water and 20% ethanol (150 ml) for 72 h. The resultant mixture underwent filtration using Whatman^®^ filter paper Grade 1 to produce the necessary extract for synthesising silver nanoparticles.

Similarly, an aqueous extract was derived from *Zingiber officinale* roots by soaking crushed ginger root (20 g) in 150 ml of 80% distilled water and 20% ethanol solution for 72 h. The resulting mixture underwent a filtration process to obtain the essential ginger extract required for silver nanoparticle synthesis.

**Fig. 1:**
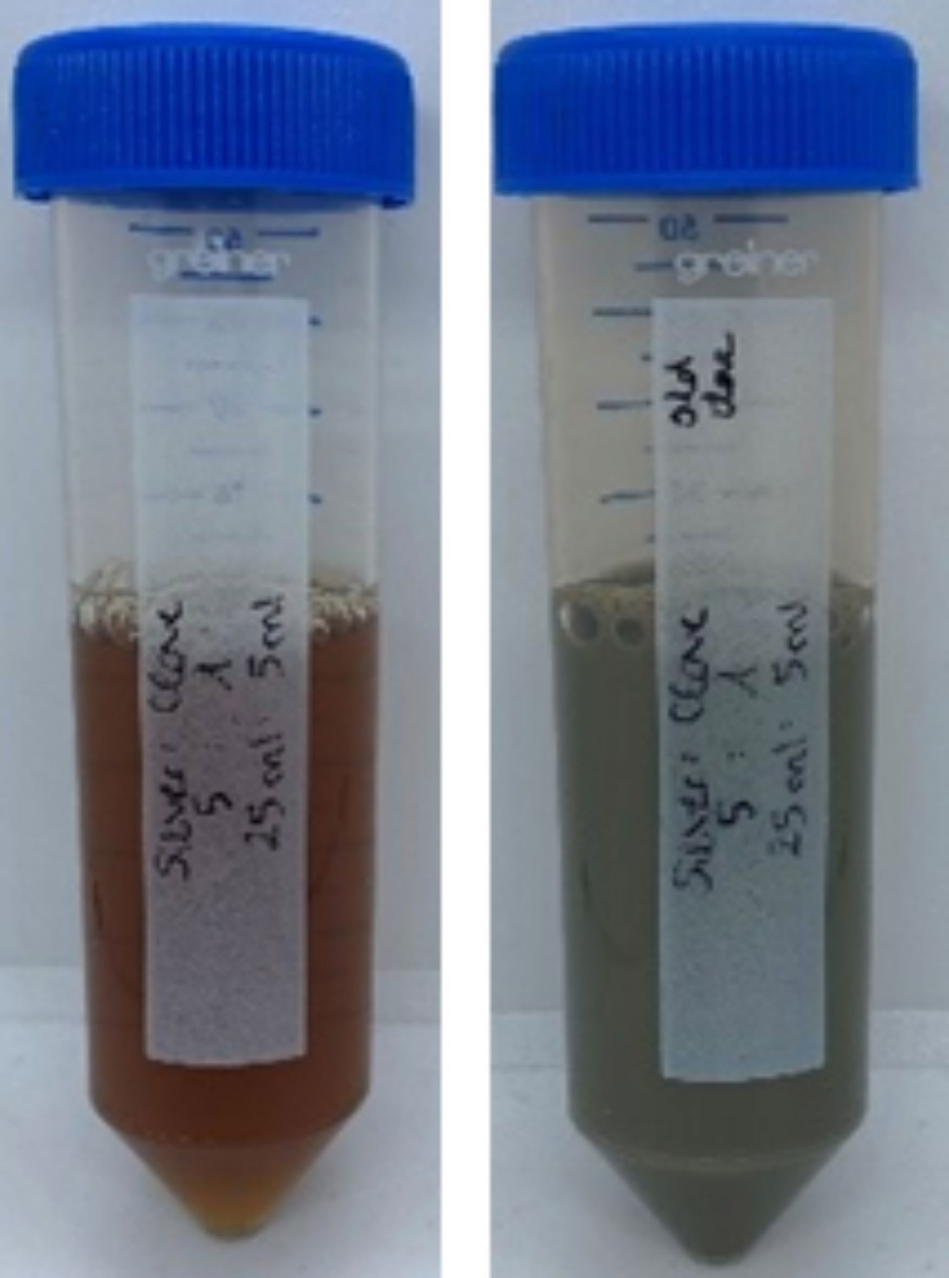
Observation of colour change in the silver nitrate solution and *Syzygium aromaticum* extract mixture after incubation confirms the green synthesis of silver nanoparticles.

Following this, the 0.2% silver nitrate solution was combined with the prepared aqueous plant extract (in a 5:1 ratio) and subjected to a 36-hour incubation period. A visible change in the solutions’ colour indicated the successful formation of silver nanoparticles (Fig. 1). These resultant mixtures of silver nitrate solution and plant extract were used to prepare two of the alginate groups^19,20^. Moreover, the 2% CHX solution was used to prepare the positive control group while distilled water was employed to prepare the negative control group (Table 2).

**Table 2:** Experimental groups and descriptions of alginate mixtures.

Experimental Groups	Description
Negative Control	Alginate mixed only with pure distilled water.
Positive Control	Alginate mixed with a 0.2% chlorhexidine solution instead of water.
*SA* + AgNPs	Alginate mixed with a solution composed of silver nitrate and cloves extract instead of water (5:1), following the manufacturer’s recommended ratios.
*ZO* + AgNPs	Alginate mixed with a solution comprising silver nitrate and ginger extract in place of water (5:1), adhering to the manufacturer’s guidelines for mixing ratios.

### Elastic recovery

**Fig. 2:**
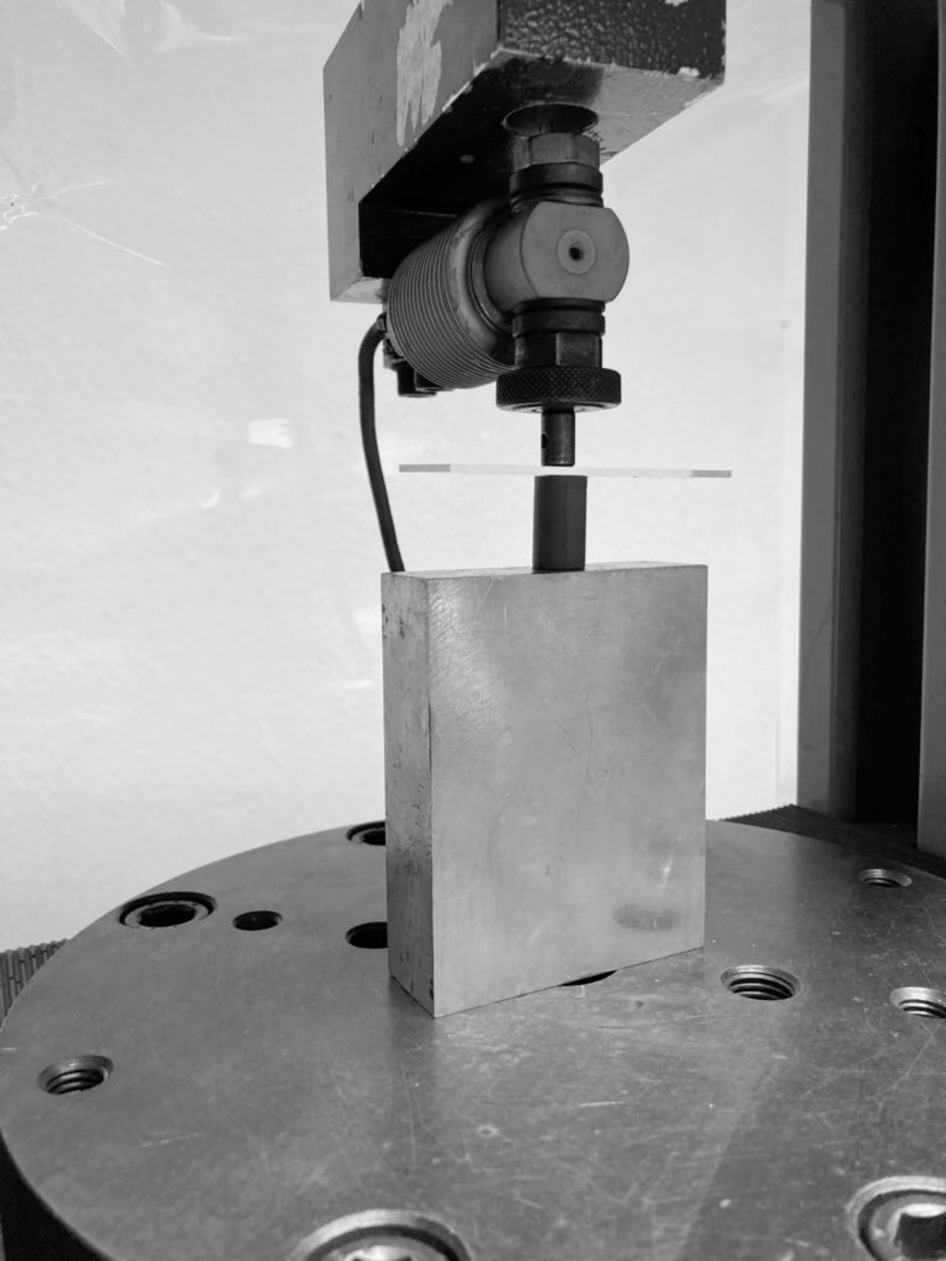
Elastic recovery specimen inside the Zwick testing machine.

The test specimens (*n* = 10) were prepared following ISO 21563:2021^21^ using a split metal mold made of stainless steel (height of 20 mm, diameter of 12.5 mm). It was additionally surrounded by a plastic stabilising ring. The freshly mixed alginate was packed inside the mold, which was thinly coated with Vaseline, and covered with a glass plate. Additionally, a 500 g weight was applied on the top to ensure proper removal of excess material and to prevent the formation of air bubbles within the mold. The set al.ginate specimen was removed from the mold and inspected for any defects or voids. After 10 min, the length of each test specimen was measured using a digital caliper (A), after which it was tested in a Zwick materials testing machine (Zwick Zmart Pro, ZwickRoell GmbH & Co. KG, Ulm, Germany) (Fig. 2). Each specimen was deformed by 20% of its initial length for 5 s. Then the load was gradually released to allow recovery from the deformation and the final length was measured (B). The change in length from the initial measurement was then calculated using the following equation to determine the percentage of elastic recovery^22^:

[(A - B/A) - 1] x 100.

### Detail reproduction

**Fig. 3:**
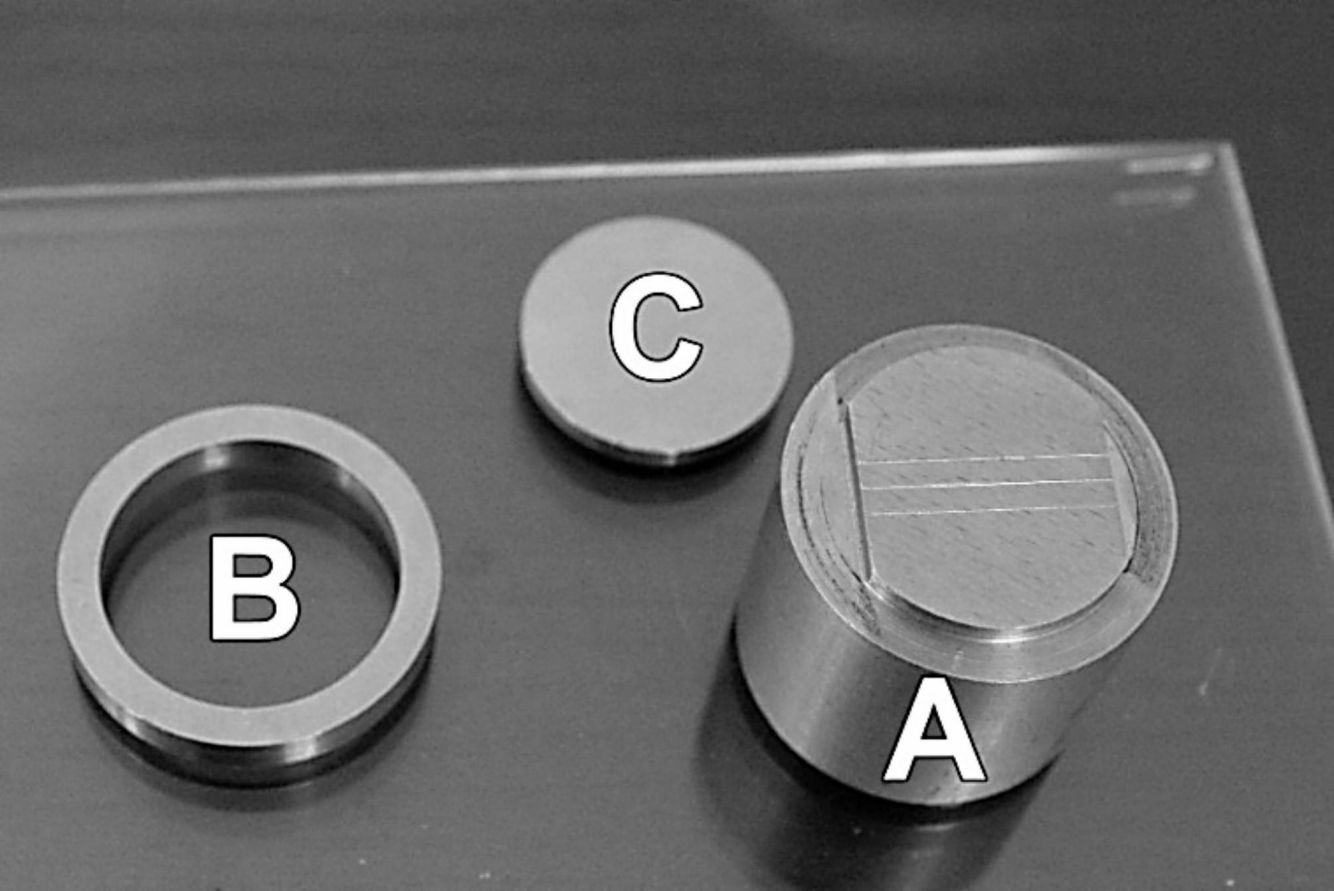
Metal mold for detail reproduction test. (**A**) Mold main body with three engraved vertical lines (25, 50 and 75 μm depth), (**B**) hollow ring, (**C**) lid cover.

To test the detail reproduction, a three-part stainless-steel mold consisting of a main body, ring and lid was used to produce the alginate specimens (*n* = 10)^19^. Three vertical lines, each 25 mm long, were engraved on the mold’s main body surface in accordance with ISO 21563:2021^21^. These lines varied in depth, measuring 20 μm, 50 μm, and 75 μm, respectively^21^ (Fig. 3). The freshly mixed alginate was placed in the space provided by fitting the hollow ring over the engraved main body of the mold. The lid was then placed on top and pressed down to cover the mold. A 500 g weight was placed on the top to allow the removal of excess material and to simulate the clinical impression-taking process. After setting, each specimen was removed carefully from the mold and examined using a light microscope (Carl Zeiss, Oberkochen, Germany) at 12-x magnification. The specimen passed the detail reproduction test if the 50 μm deep line was reproduced in its entire length in the alginate, for which only a “yes” or “no” decision was made based on this criterion^22^.

### Tear strength

**Fig. 4:**
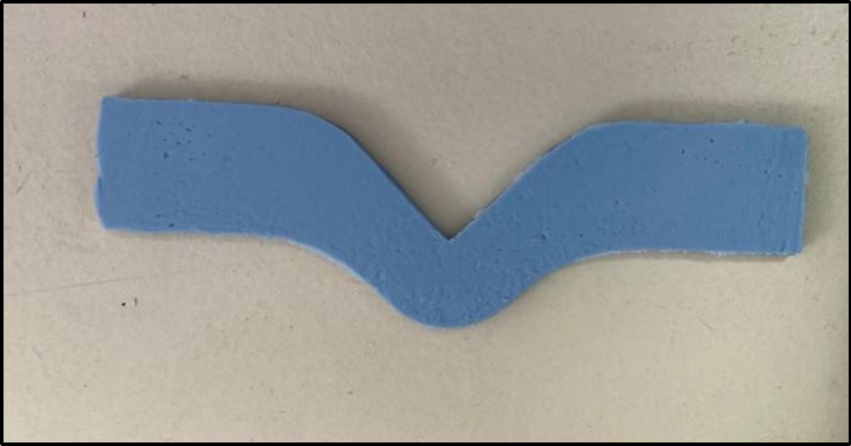
Tear strength specimen.

The Zwick materials testing machine was also used for testing the tear strength of the set al.ginate. A plastic mold with 10 cm in length, 2 cm in width, and 4 mm in height was 3D printed (Renkforce RF100, Conrad, Hirschau, Germany) following ISO 21563:2021to be used for tear strength assessment^21^ (Fig. 4). Test specimens (*n* = 10) were prepared from freshly mixed alginate, filled in the plastic mold, and covered with a glass plate with a 500 g weight placed on the top to ensure uniform sample thickness with a smooth surface^19,20^.

Each set specimen was inspected for defects and left to rest for 10 min before the thickness of the sample at the tearing point was determined using a digital caliper. In a Zwick testing machine, each specimen was subjected to a tensile load at a crosshead speed of 500 mm/min until the specimen experienced tear failure. The force required to rupture the alginate was recorded and used, along with the specimen thickness, to determine the tear strength as per the following equation:

Tear strength in N/mm = tearing force\thickness of the specimens.

### Surface roughness of the gypsum model

**Fig. 5:**
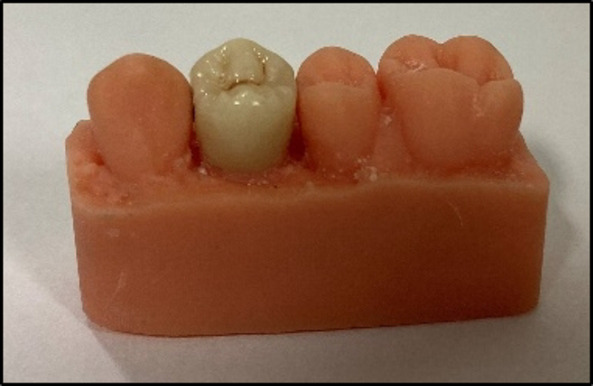
Plastic model for impression taking to produce plaster specimens.

The surface roughness of plaster models was analysed using the optical profilometer MicroSpy (FRT GmbH, Bergisch-Gladbach, Germany). A small plastic model of a canine, two premolars, and a molar were replicated with each alginate group (*n* = 10) using a plastic perforated tray of a convenient size (Fig. 5). The produced impressions were then poured with type IV superhard plaster to produce the plaster test models. According to the manufacturer´s instructions 20 g of the plaster powder was mixed with 100 ml distilled water, first briefly by hand and then mechanically for 30 s under vacuum.

The alginate impressions were poured on a vibrating plate and after 30 min setting time, the plaster models were taken out of the impressions. A template made from addition-curing silicone, featuring a 3 mm x 3 mm window, was employed to establish a reference area, ensuring consistent examination of each specimen within the same region. Using this template, the test area was marked on each specimen on the base, specifically at the level of the first premolar. The roughness value (Ra-value) was determined five times in each test field, one measurement in each corner and one measurement in the middle of the field and the mean was calculated. The software FRT III, which is associated with the profilometer, was used to evaluate the measurements regarding the surface roughness^23^.

### Statistical analysis

The software GraphPad Prism 10.1.2 was used to analyse the results statistically. First the mean and standard deviation were determined for each test. To check the normality, Shapiro-Wilk normality test was conducted. A one-way analysis of variance (ANOVA) test was used to compare between groups, which was followed by Tukey´s post hoc test for pairwise comparison if the ANOVA test was significant. Kruskal-Wallis- and Dunn post hoc test were applied when data did not show normal distribution. For each test, the level of significance was set at α of 0.05.

## Results

### Elastic recovery

**Fig. 6:**
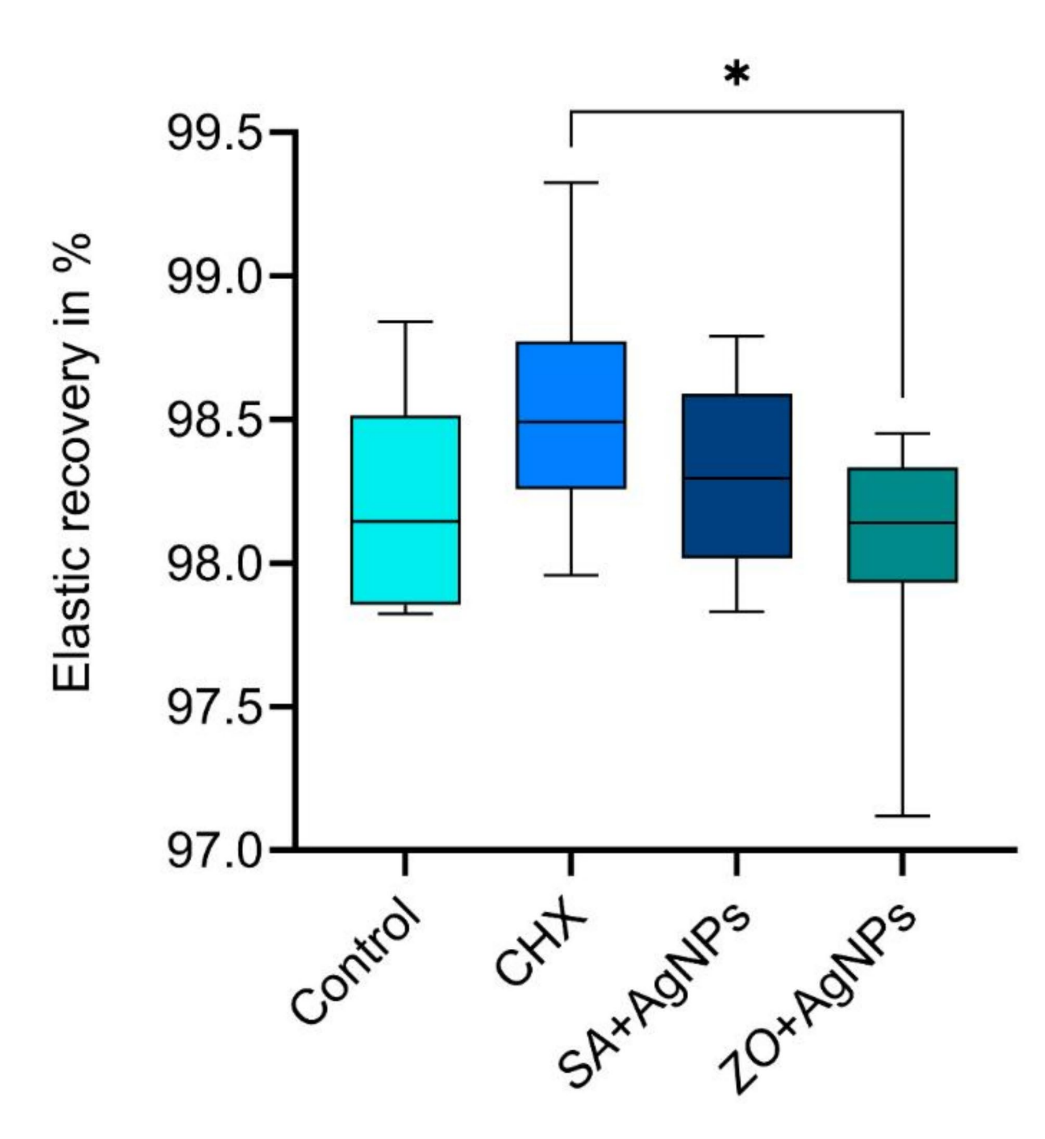
Box plot representing the elastic recovery in % of the four tested alginate materials.

The means and standard deviations of percentage recovery from deformation are represented in Fig. 6. Tukey’s post hoc test showed a significant difference only between the CHX with the highest mean elastic recovery (98.5 ± 0.4%) and the *ZO* + AgNPs group with the lowest mean elastic recovery (98.0 ± 0.4%; p-value = 0.04). The control group had a mean elastic recovery of 98.2 ± 0.4%, which was insignificantly different from the *SA* + AgNPs (98.3 ± 0.3%) and the other two modified groups.

### Detail reproduction

All specimens of all tested groups reproduced the entire length of the 50 μm line. They achieved the requirement in the ISO 21563:2021 and passed the test.

### Tear strength

**Fig. 7:**
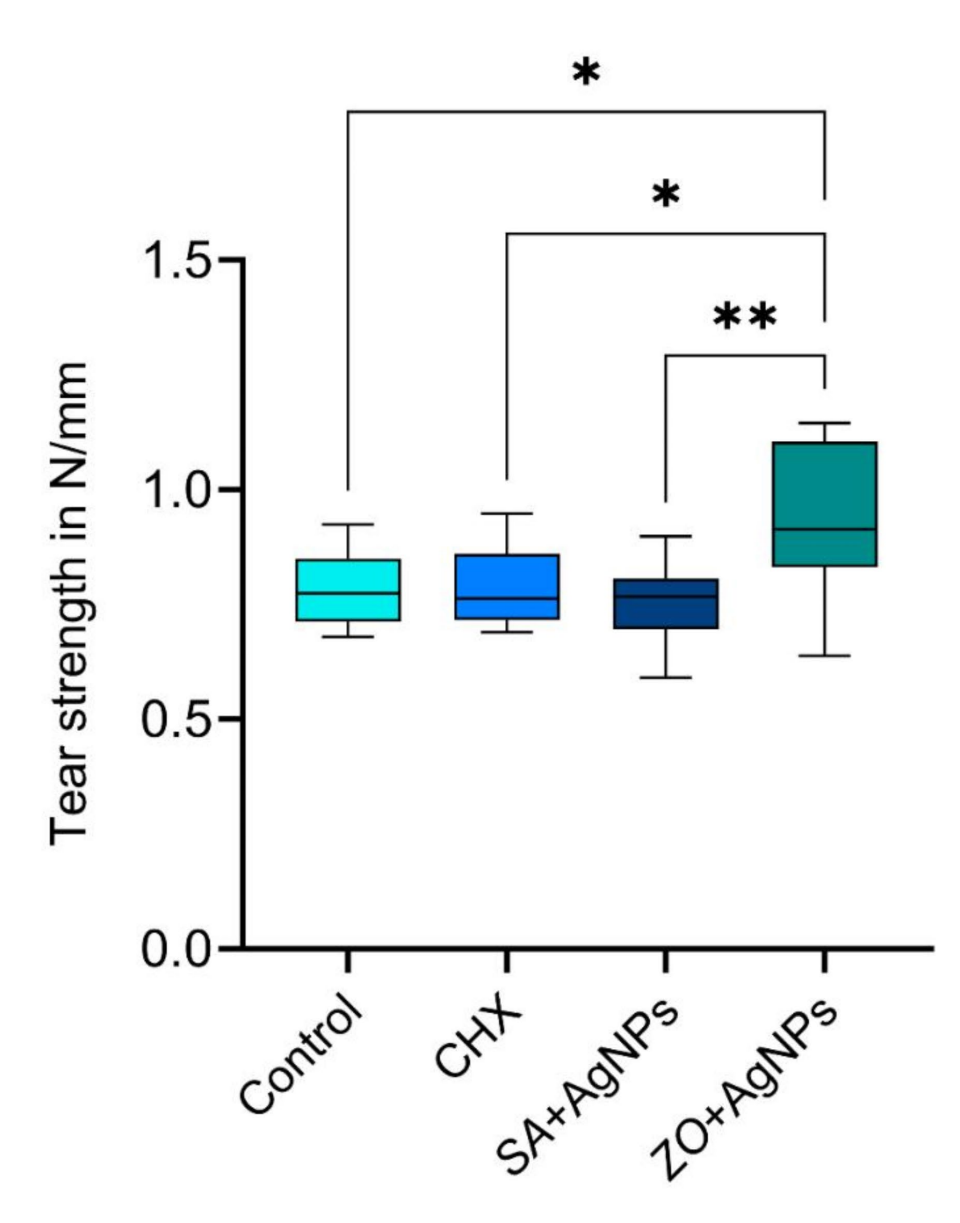
Box plot representing the tear strength in N/mm of the four tested alginate materials.

The means and standard deviations of the tear strength are presented in Fig. 7. The results were normally distributed and so an ANOVA was applied, which indicated that there was a statistically significant difference in the tear strength of the four tested groups (p-value = 0.005). The control group had a mean tear strength of 0.79 ± 0.09 N/mm, which was insignificantly different from the *SA* + AgNPs (0.76 ± 0.09 N/mm) and CHX (0.79 ± 0.09 N/mm). The *ZO* + AgNPs group showed the highest tear strength (0.94 ± 0.17 N/mm), which was significantly higher than the three other tested groups.

### Surface roughness of gypsum cast

**Fig. 8:**
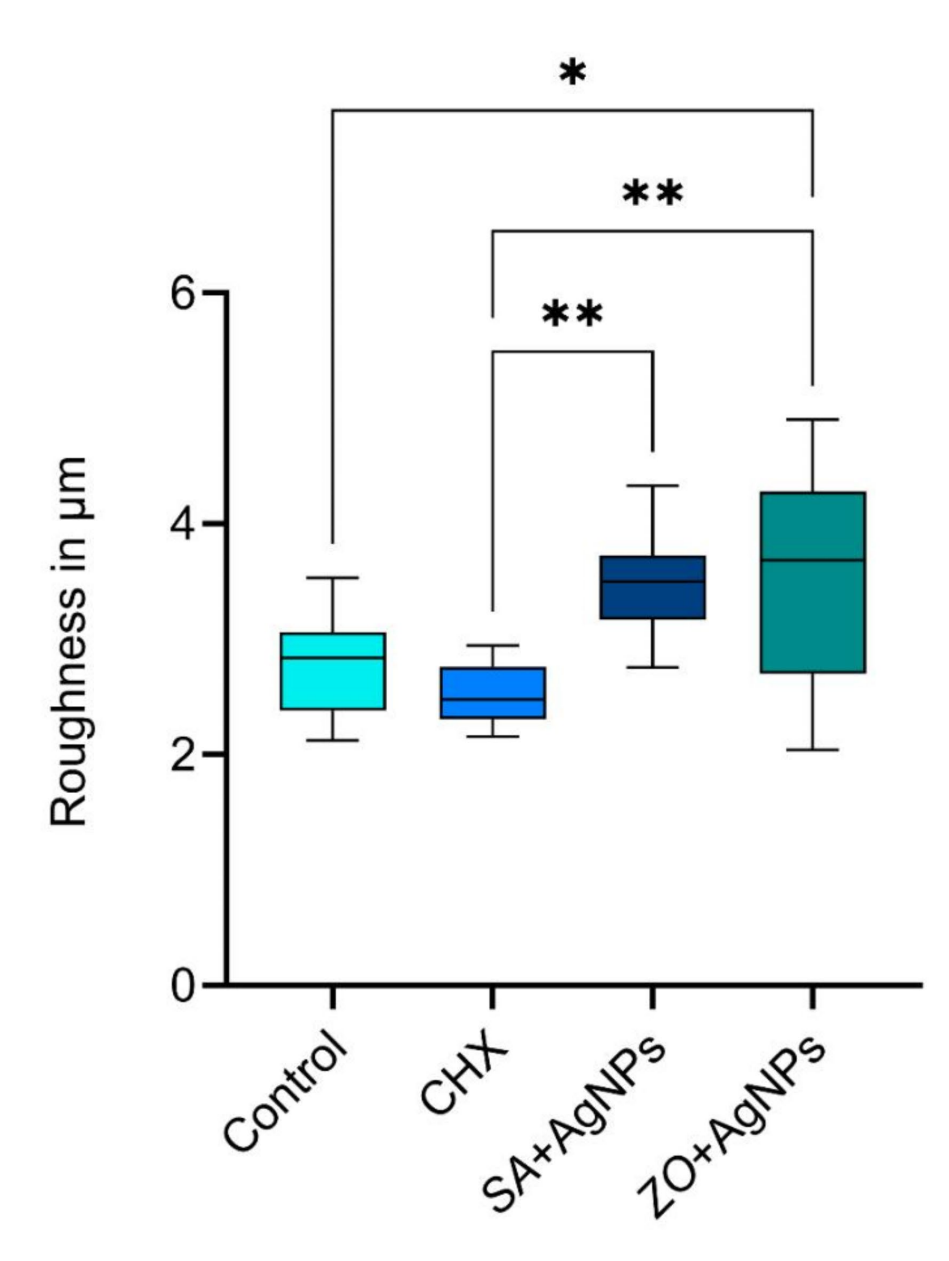
Box plot representing the surface roughness of gypsum model in µm of the four tested alginate materials.

Figure 8 illustrates the means and standard deviations of the surface roughness of the gypsum cast. The p-value of the ANOVA was < 0.001, which displayed that there was a statistically significant difference in the roughness of the four tested groups. The CHX group had a mean surface roughness of 2.53 ± 0.25 μm, which was significantly lower than both AgNPs groups. The *SA* + AgNPs modification (3.48 ± 0.45 μm) showed no significant difference compared to the control group (2.79 ± 0.44 μm) or the *ZO* + AgNPs (3.53 ± 0.96 μm). *ZO* + AgNPs was significantly rougher than the control group.

## Discussion

Dental alginate serves as a medium for the transmission of microorganisms, potentially leading to cross-infection between dental clinicians and laboratory technicians. While rinsing impressions with water after removal from the mouth helps reduce the microbial load of alginate, it does not effectively disinfect the dental impression material. Therefore, additional methods are always employed to ensure effective eradication of oral pathogens^24^. The use of natural plant extracts in modifying dental materials is undergoing thorough investigation in contemporary research. This emerging approach holds significant importance for several reasons such as the inherent antimicrobial properties of phytochemical compounds, providing a safe and sustainable alternative to chemical agents and reducing the risk of adverse reactions^10^.

Moreover, their eco-friendly nature aligns with global efforts towards sustainable practices, and their diverse bioactive compounds effectively combat microorganisms, potentially enhancing dental materials’ antimicrobial properties while minimising environmental impact^25^. Therefore, in the present study, elastic recovery, tear strength, and surface roughness of gypsum cast of alginates mixed with three different disinfectant solutions instead of pure water were tested (0.2% chlorhexidine solution (CHX group), aqueous extracts of *Syzygium aromaticum* (*SA* + AgNPs) and *Zingiber officinale* (*ZO* + AgNPs) mixed with green-synthesised silver nanoparticles.

In our previous investigation, the mentioned modified groups underwent testing for antimicrobial activity against *S. mutans*, MRSA, MSSA, and *C. albicans* using the Agar Well Diffusion assay. In addition, we analysed the impact of the modifications on the dimensional stability of the dental alginate. Both green silver nanoparticle groups (*SA* + AgNPs and *ZO* + AgNPs) demonstrated significant antimicrobial activity against *S. mutans*, MRSA, MSSA compared to the control group, and their efficacy was comparable to CHX, the gold standard antimicrobial agent in dentistry^18^. Additionally, only the *SA* + AgNPs green group exhibited effectiveness against *C. albicans* compared to all tested groups. Furthermore, in the same previous study, we observed that the dimensional accuracy of the modifications with green synthesised AgNPs remained unaffected^18^.

Consequently, having confirmed positive antimicrobial activity and dimensional accuracy outcomes, it was the aim of this study to further explore the effects of this approach on additional physical and mechanical properties of alginate. Elastic recovery is a determining property of an impression material, signifying its ability to regain its original shape after being deformed. The higher the elastic recovery, the lower the amount of permanent deformation, resulting in a more accurate cast^22^. In the current study, none of the three modified groups showed a significantly altered elastic recovery in comparison to the negative control group. All tested groups met the minimal requirement for elastic recovery for alginate impression material given in the ISO 21563:2021, which is set at 95%. The results are in agreement with Ginjupalli et al.^26^, who examined the effect of incorporating different particle sizes and concentrations of silver nanoparticles into alginate on various properties, including permanent deformation^26^. Moreover, Singer et al. showed that incorporating silver nanoparticles as a natural disinfectant could be a promising approach to boost antimicrobial effectiveness without compromising elastic recovery^19^.

Achieving a detailed and accurate replica of oral hard and soft tissues necessitates precise detail reproduction by the impression material. This property is mainly determined by the viscosity and flow characteristics of the material, among other factors^27^. It has been established that lower viscosity in the material leads to improved flow, resulting in more accurate reproduction of fine details^27^.

In our study, all specimens from all groups precisely and consistently reproduced the entire length of the 50-µm line of the mold, meeting the ISO requirement. This can be explained by the non-altered flowability and viscosity of the unset al.ginate material despite using other mixing solutions than water^28^. Correspondingly, Omidkhoda et al. concluded in their study, that the incorporation of silver nanoparticles into alginate material has no negative effect on detail reproduction^29^.

The tear strength of an impression material is another crucial property, describing the ability of the material to resist tearing. Mainly in areas with undercuts, thin interproximal areas, and for a thin material thickness, sufficient tear strength is required to ensure the alginate withstands tearing force and rupture during impression removal from the mouth^30^. As per ISO 21563:2021 standards^21^, alginate impression material should possess a minimum tear strength of 0.38 N/mm. Remarkably, all examined groups not only met this criterion but also demonstrated enhanced tear strength. Notably, the *ZO* + AgNPs group was significantly higher than the other three groups, exhibiting the highest tear strength recorded at 0.94 ± 0.17 N/mm.

The degree of polymerization and crosslinking in set al.ginate material plays a crucial role in determining properties such as strength, viscosity, elasticity, and recovery from deformation^31,32^. While increased polymerization can enhance strength, it may also lead to higher viscosity, which can adversely affect elasticity, flowability, and detail reproduction^33^. The size and distribution of nanoparticles incorporated into the alginate are also critical; optimal dispersion enhances mechanical reinforcement, while agglomeration can weaken the material. Furthermore, chemical interactions between the nanoparticles and the alginate matrix can alter polymerization and crosslinking dynamics, influencing overall stability^34,35^.

In this study, the *ZO* + AgNPs group exhibited significantly increased tear strength, likely due to a greater degree of crosslinking within the alginate. Importantly, this enhancement did not adversely affect elastic recovery, and detail reproduction. These findings suggest that the modifications introduced by the ginger nanoparticles improve crosslinking without significantly increasing viscosity, thereby maintaining the material’s mechanical properties and ability for fine detail reproduction. Notably, the *ZO* + AgNPs group demonstrated less agglomeration compared to the *SA* + AgNPs group in our previous study^18^, further contributing to its superior tear strength. This may be attributed to the unique reinforcing properties of the ginger extract and its favourable interactions with the alginate polymer chains.

In the literature, no clinically accepted values for surface roughness are specified. Notably, both AgNPs groups exhibited significantly rougher surfaces compared to the CHX group. However, there was no notable difference between the *SA* + AgNPs group and the control group. While the surface roughness of the *ZO* + AgNPs group was slightly elevated compared to the control group, the difference in mean values was less than 1 μm. Therefore, it might be argued that it is not clinically significant, especially when considering the use of alginate impressions in dentistry. Rentzia et al.^35^ explored how different disinfection solutions affected the surface quality of gypsum models (smooth and rough areas) from an irreversible hydrocolloid impression material. While the smooth area remained unaffected, longer immersion times led to increased surface roughness in the rough region^36^.

One of the limitations of this study was that the tests were performed under in-vitro conditions using molds and without the presence of saliva. This omission may influence factors such as the detail reproduction of the alginate or the tear strength, as entrapment of saliva inside the material while setting could lead to voids. Another factor is that the alginate was manually mixed, a method that closely mirrors the clinical practice in dental offices. However, manual mixing may lead to a higher failure rate and differences among the samples. This consideration highlights the need for further investigation into the impact of manual mixing on study outcomes.

## Conclusions

Based on this study and the previous work, it can be concluded that silver nanoparticles synthesised and mixed with plant extracts from *Syzigium aromaticum* and *Zingiber officinale* could be very effective agents for intrinsic antimicrobial modification of dental alginate. They showed efficacy against *S. mutans*, MRSA, MSSA and *C. albicans*, without affecting the mechanical and physical properties of the alginate adversely. All groups exhibited an even better elastic recovery and tear strength than the ISO required and the requirement for detail reproduction was met. Regarding the roughness of the gypsum specimens, although the *ZO* + AgNPs group revealed a significantly rougher gypsum surface than the control group, the difference is not considered clinically relevant. Furthermore, the use of green-synthesized silver nanoparticles (AgNPs) to modify alginate offers a cost-effective alternative to traditional chemical synthesis methods, significantly reducing material expenses and processing costs. Additionally, the eco-friendly approach not only lowers production costs but also minimizes environmental impact, making it a sustainable choice for dental applications.

## Data Availability

All data generated in the current study or analysed during this study are included in this published article.
